# *In vitro* susceptibility of contagious ovine digital dermatitis associated *Treponema* spp. isolates to antimicrobial agents in the UK

**DOI:** 10.1111/vde.12269

**Published:** 2015-10-19

**Authors:** Joseph W. Angell, Simon R. Clegg, Leigh E. Sullivan, Jennifer S. Duncan, Dai H. Grove‐White, Stuart D. Carter, Nicholas J. Evans

**Affiliations:** ^1^ Department of Epidemiology and Population Health Institute of Infection and Global Health The University of Liverpool, Leahurst Campus Neston Wirral CH64 7TE UK; ^2^ Department of Infection Biology Institute of Infection and Global Health The University of Liverpool, Liverpool Science Park IC2 146 Brownlow Hill Liverpool L3 5RF UK

## Abstract

**Background:**

Contagious ovine digital dermatitis (CODD) is an important cause of infectious lameness in sheep in the UK and Ireland and has a severe impact on the welfare of affected individuals. The three treponemal phylogroups *Treponema medium/Treponema vincentii*‐like, *Treponema phagedenis*‐like and *Treponema pedis* spirochaetes have been associated with clinical CODD lesions and are considered to be a necessary cause of disease. There are scant data on the antimicrobial susceptibility of the treponemes cultured from CODD lesions.

**Objective:**

The aim of this study was to determine *in vitro* the miniumum inhibitory concentration/ minimum bactericidal concentration (MIC/MBC) of antimicrobials used in the sheep industry for isolates of the three CODD associated treponeme phylogroups *T. medium/T. vincentii*‐like, *T. phagedenis*‐like and *T. pedis*.

**Animals:**

Twenty treponeme isolates; from 19 sheep with clinical CODD lesions.

**Methods:**

A microdilution method was used to determine *in vitro* the MIC/MBC of 10 antimicrobial agents for 20 treponeme isolates (five *T. medium/T. vincentii*‐like, 10 *T. phagedenis*‐like and five *T. pedis*). The antimicrobials tested were penicillin G, amoxicillin, oxytetracycline, tilmicosin, lincomycin, spectinomycin, tylosin, tildipirosin, tulathromycin and gamithromycin.

**Results:**

The treponeme isolates tested showed low MICs and MBCs to all 10 antimicrobials tested. They were most susceptible to gamithromycin and tildipirosin (MIC90: 0.0469 mg/L), and were least susceptible to lincomycin, spectinomycin and oxytetracycline (MIC90: 48 mg/L, 24 mg/L and 3 mg/L, respectively).

**Conclusions:**

These data are comparable to *in vitro* antimicrobial susceptibility data for treponemes cultured from bovine digital dermatitis lesions. Dependent on local licensing, penicillin and tilmicosin appear to be the best candidates for future *in vivo* studies.

## Abstract 2

**Background** – Contagious ovine digital dermatitis (CODD) is an important cause of infectious lameness in sheep in the UK and Ireland and has a severe impact on the welfare of affected individuals. The three treponemal phylogroups *Treponema medium/Treponema vincentii*‐like, *Treponema phagedenis*‐like and *Treponema pedis* spirochaetes have been associated with clinical CODD lesions and are considered to be a necessary cause of disease. There is scant data on the antimicrobial susceptibility of the treponemes cultured from CODD lesions. **Objective** – The aim of this study was to determine *in vitro* the MIC/MBC of antimicrobials used in the sheep industry for isolates of the three CODD‐associated treponeme phylogroups *T. medium/T. vincentii*‐like, *T. phagedenis*‐like and *T. pedis*.**Conclusions** – These data are comparable to *in vitro* antimicrobial susceptibility data for treponemes cultured from bovine digital dermatitis lesions. Dependent on local licensing, penicillin and tilmicosin appear the best candidates for future *in vivo* studies.

## Résumé

**Contexte:**

La dermatite digitale contagieuse ovine (CODD) est une cause infectieuse importante de boiterie chez le mouton au Royaume Uni et en Irlande et a un impact sévère sur le bien être des sujets atteints. Les trois phylogroupes de spirochaetes *Treponema medium/Treponema vincentii*‐like, *Treponema phagedenis*‐like et *Treponema pedis* ont été associés avec des lésions cliniques de CODD et sont considérés comme les causes obligatoires de la maladie. Il existe peu de données sur les sensibilités antimicrobiennes des tréponèmes issus des lésions de CODD.

**Objectif:**

Le but de l’étude était de déterminer la concentration minimale inhibitrice *in vivo* et la concentration bactéricide minimale (MIC/MBC) des antimicrobiens utilisés dans l'industrie ovine pour les souches des trois phylogroupes de tréponèmes associés au CODD *T. medium/T. vincentii‐like, T. phagedenis*‐like et *T. pedis*.

**Sujets:**

Vingt souches de tréponème, issues de 19 moutons présentant des lésions de CODD.

**Méthodes:**

Une méthode de microdilution a été utilisée pour déterminer le MIC/MBC *in vitro* de 10 agents antimicrobiens pour 20 souches de tréponèmes (cinq *T. medium/T. vincentii‐*like, 10 T. *phagedenis*‐like et cinq *T. pedis*). Les antimicrobiens testés étaient la pénicilline G, l'amoxicilline, l'oxytétracycline, la tilmycosine, la lincomycine, la spectinomycine, la tylosine, la tildipirosine, la tulathromycine et la gamithromycine.

**Résultats:**

Les souches de tréponèmes testées ont montré de faibles MIC et MBC à tous les 10 antimicrobiens. Elles étaient plus sensibles à la gamithromycine et la tildipirosine (MIC90: 0.0469 mg/L), et étaient au moins sensibles à la lincomycine, la spectinomycine et l'oxytétracycline (MIC90: 48, 24 and 3 mg/L, respectivement).

**Conclusions:**

Ces données sont comparables aux données de sensibilité aux antimicrobiens *in vitro* pour les tréponèmes issus de lésions podales des bovins. En fonction des autorisations locales, la pénicilline et la tilmicosine semblent être les meilleures options pour de prochaines études *in vivo*.

## Resumen

**Introducción:**

la dermatitis contagiosa digital ovina (CODD) es una causa importante de de cojera infecciosa en ovejas en el Reino Unido e Irlanda y tiene un impacto severo en la salud de los individuos afectados. Los tres filogrupos de espiroquetas de Treponema *Treponema medium*/*Treponema similar a T. vincentii*, Treponema similar a *T. phagenedis* y *Treponema pedis* han sido asociados con lesiones clínicas de CODD y están considerados como una causa necesaria de enfermedad. Hay pocos datos acerca de la susceptibilidad antimicrobiana de los cultivos de Treponema obtenidos de lesiones de CODD.

**Objetivo:**

el propósito de este estudio fue determinar la concentración inhibitoria mínima/concentración mínima bactericida (MIC/MBC) *in vitro* de antimicrobianos utilizados en la industria ovina frente a asilados de los tres filogrupos de Treponema asociados con CODD *Treponema medium*/Treponema similar *a T. vincentii*, Treponema similar a *T. phagenedis* y *Treponema pedis*.

**Animales:**

20 aislados de Treponema de 19 ovejas con lesiones clínicas de CODD.

**Métodos:**

se utilizó un método de microdilución para determinar la MIC/MBC *in vitro* de 10 agentes antimicrobianos frente a 20 aislados de Treponema (cinco *Treponema medium*/*Treponema similar a T. vincentii*, diez de Treponema similar a *T. phagenedis* y cinco de *Treponema pedis*). Los antimicrobianos probados fueron penicilina G, amoxicilina, oxitetraciclina, tilmicosina, lincomicina, espectinomicina, tilosina, tildipirosina, tulatromicina y gamitromicina.

**Resultados:**

los asilados de Treponema probados mostraron bajas MICs y MBCs en los 10 antimicrobianos probados. Fueron más susceptibles a gamitromicina y tildipirosina (MIC90: 0,0469 mg/L) y fueron menos susceptibles a lincomicina, espectinomicina y (MIC90: 48, 24, y 3 mg/L respectivamente.

**Conclusiones e importancia clínica:**

estos datos son comparables a la susceptibilidad antimicrobiana *in vitro* de Treponemas cultivados de lesiones dermales digitales bovinas. Dependiendo de las licencias locales, la penicilina y tilmicosina pueden ser los mejores candidatos para futuros estudios *in vivo*.

## Zusammenfassung

**Hintergrund:**

Die kontagiöse ovine digitale Dermatitis (CODD) ist eine wichtige Ursache für infektiöse Lahmheit bei Schafen im Vereinigten Königreich und in Irland und hat gravierende Einflüsse auf das Wohlbefinden der betroffenen Tiere. Die drei Phylogruppen der Treponema, *Treponema medium/Treponema vincentii*‐like, *Treponema phagedenis*‐like und *Treponema pedis* Spirochäten sind mit klinischen CODD Veränderungen in Zusammenhang gebracht worden und werden als notwendige Ursache für eine Erkrankung gesehen. Es gibt nur spärliche Daten über die antimikrobielle Empfindlichkeit der Treponema, die aus CODD Veränderungen kultiviert wurden.

**Ziel:**

Das Ziel dieser Studie war es, die minimale inhibitorische *in vitro* Konzentration/minimale bakterizide Konzentration (MIC/MBC) der Antibiotika zu bestimmen, die in der Schafindustrie für Isolate der CODD verursachenden Treponema Phylogruppen, *T. medium/T. vincentii‐*like, *T. phagedenis*‐like und *T. pedis* Verwendung finden.

**Tiere:**

Zwanzig Treponema Isolate; von 19 Schafen mit klinischen CODD Veränderungen.

**Methoden:**

Eine Mikrodilutionsmethode wurde angewendet, um die *in vitro* MIC/MBC von 10 Antibiotika für 20 Treponema Isolate (fünf *T. medium/T. vincentii*‐like, 10 *T. phagedenis*‐like und fünf *T. pedis*) zu bestimmen.

**Ergebnisse:**

Die getesteten Treponema Isolate zeigten niedrige MICs und MBCs gegenüber allen 10 getesteten Antibiotika. Sie waren gegenüber Gamithromycin und Tildipirosin empfindlicher (MIC90: 0,0469 mg/L, und waren am wenigsten empfindlich gegenüber Lincomycin, Spectinomycin und Oxytetracyclin (MIC90: 48, 24 bzw. 3 mg/L).

**Schlussfolgerungen:**

Diese Daten sind mit den *in vitro* antimikrobiellen Empfindlichkeitsdaten für Treponema, die aus bovinen digitalen Hautveränderungen kultiviert wurden, vergleichbar. Je nach lokaler Zulassung, scheinen Penicillin und Tilmicosin zukünftig die besten Kandidaten für *in vitro* Studien zu sein.

## 要約

**背景:**

伝染性羊趾皮膚炎(CODD)はUKおよびアイルランドにおける羊の感染性跛行の重要な原因の1つであり、罹患した個体の健康に重大な影響を与える。3種類のトレポネーマの系統群である*Treponema medium/Treponema vincentii*‐like、*Treponema phagedenis*‐likeおよび*Treponema pedis*が臨床的なCODD病変と関与し、疾患に必須の原因と考えられている。CODD病変から培養されたトレポネーマの抗菌剤感受性に関する情報はほとんどない。

**目的:**

この研究の目的はCODDに関連した3つのトレポネーマ統計群である*T. medium/T. vincentii‐like, T. phagedenis*‐like および *T. pedis*.の分離菌に対して養羊業界で使用する抗菌剤の*in vitro*の最小阻止濃度/最小細菌濃度(MIC/MBC)を明らかにすることである。

**供与動物:**

臨床的にCODDの症状を示す19頭の羊から分離した20種類のトレポネーマ分離菌

**方法:**

20種類のトレポネーマ分離菌(5つの*T. medium/T. vincentii‐*like, 10つの T. *phagedenis*‐likeおよび5つの*T. pedis*)に対する10種類の抗菌薬の*in vitro*の最小阻止濃度/最小細菌濃度(MIC/MBC)を微量希釈法を用いて決定した。検査した抗菌薬はペニシリンG、アモキシシリン、オキシテトラサイクリン、チルミコシン、リンコマイシン、スペクチノマイシン、タイロシン、チルジピロシン、ツラスロマイシンならびにガミスロマイシンであった。

**結果:**

検査を行ったトレポネーマ分離菌は、検査した10種類すべての抗菌剤に対して低いMICおよびMBCを示した。それらの菌はガミスロマイシンおよびチルジピロシンで最も感受性が高く(MIC90は0.0469 mg/L)、リンコマイシン、スペクチノマイシン、およびテトラサイクリンに対しては最も感受性が低かった(それぞれのMIC90は48 mg/L, 24 mg/L and 3 mg/L)。

**結論:**

これらの情報はウシの趾皮膚炎病変から分離したトレポネーマの*in vitro*の抗菌剤感受性データと一致した。地域で承認されている薬にもよるが、ペニシリンおよびチルミコシンはさらなる*in vivo*研究のための最もよい候補となるかもしれない。

## 摘要

**背景:**

绵羊传染性趾炎(CODD),是英国和爱尔兰绵羊感染性跛行的一个重要原因,它严重影响患病动物的生活质量。*Treponema medium/*文氏密螺旋体、梅毒密螺旋体和足密螺旋体这三种密螺旋体型,是引起CODD临床病变的必要病因。CODD病灶的密螺旋体培养,其药敏资料不完整。

**目的:**

研究的目的,是确定引起CODD的三种菌株:*Treponema medium/*文氏密螺旋体、梅毒密螺旋体和足密螺旋体,其抗菌剂的体外最小抑菌浓度和最小杀菌浓度(MIC/MBC)。

**动物:**

从具有CODD临床病变的10只绵羊上分离20份密螺旋体菌株。

**方法:**

使用微稀释法确定10种抗菌剂分别对20份密螺旋体菌株(5株 *T. medium/T. vincentii‐*like, 10株 T. *phagedenis*‐like 和 5株*T. pedis*)的体外MIC/MBC。参与检测的抗菌剂分别为青霉素G、阿莫西林、土霉素、替米考星、林可霉素、大观霉素、泰勒菌素、泰地罗斯、泰拉霉素和加米霉素。

**结果:**

密螺旋体的菌株测试显示,10种抗菌药物均有较低的MICs和MBCs。最敏感的抗菌药物为加米霉素和泰地罗斯(MIC90: 0.0469 mg/L),最不敏感的为林可霉素、大观霉素和土霉素(MIC90分别为 48 mg/L、24 mg/L 和3 mg/L)。

**总结:**

从牛科动物趾炎病变处取密螺旋体培养,测得并比较体外药敏数据。基于当地批准文号,青霉素和替米考星将可能成为未来体内试验的最佳候选药物。

## Introduction

Contagious ovine digital dermatitis (CODD) is a cause of infectious lameness in sheep in the UK and Ireland and has been shown to have a severe impact on the welfare of affected individuals.^1^ Recent surveys have shown that CODD may affect approximately 35% of flocks in the UK; while on‐farm prevalence is typically low, it may affect up to 50% of the flock at any one time.^1^

Information about the microbial flora of CODD lesions is limited, although the bovine digital dermatitis (BDD) associated treponemes *Treponema medium/T. vincentii*‐like, *Treponema phagedenis*‐like and *Treponema pedis* are currently considered to be a necessary cause of disease.^1^ The recent characterization of treponemes associated with CODD demonstrated the presence of at least one BDD phylotype present in all 58 lesions studied, whereas these were totally absent from all healthy sheep foot tissues.^2^

There has been a wide range of empirically chosen treatments employed in clinical cases such as parenteral oxytetracycline and topical tylosin,^3^ with only one randomized controlled trial conducted comparing parenteral amoxicillin and simultaneous topical chlortetracycline with topical chlortetracycline alone.^4^

As with CODD, the successful treatment of BDD has remained problematic with many farms adopting management and control strategies as opposed to affecting a cure.^5^ In order to inform the development of effective therapeutic strategies for clinical cases of CODD, a greater understanding is required of the susceptibility of the treponemes found in CODD lesions to antimicrobials currently available for use in sheep.

The aim of this study was to determine the minimum inhibitory concentration (MIC) and minimum bactericidal concentration (MBC) of a panel of antimicrobials for representatives from each of the three treponeme phylogroups cultured as pure isolates from clinical CODD lesions.

## Materials and methods

### Bacterial isolates

Twenty treponeme isolates from CODD lesions from 19 sheep from six farms in England, Wales and Northern Ireland were used (Table 1). Included were five isolates from the *T. medium/T. vincentii*‐like group, 10 isolates from the *T. phagedenis*‐like group and five isolates from the *T. pedis* group.

**Table 1:** Treponemes tested for susceptibility to antimicrobial agents

Strain no.	Strain	Isolation date	UK Location	Nearest related organism^a^
1	G1F7C5	07/2013	Conwy	*Treponema medium/Treponema vincentii*
2	G1F9C27	07/2013	Conwy	*Treponema medium/Treponema vincentii*
3	G1OV11	08/2009	Gloucester	*Treponema medium/Treponema vincentii*
4	G2S2R	02/2009	Cheshire	*Treponema medium/Treponema vincentii*
5	ST27	07/2013	Conwy	*Treponema medium/Treponema vincentii*
6	3F2	02/2014	Anglesey	*Treponema phagedenis*
7	C2F	06/2009	Gloucestershire	*Treponema phagedenis*
8	G2S4F	02/2009	Cheshire	*Treponema phagedenis*
9	G2F3C12	07/2013	Conwy	*Treponema phagedenis*
10	G13F3	02/2014	Denbighshire	*Treponema phagedenis*
11	G2SL1	05/2014	Anglesey	*Treponema phagedenis*
12	G23F1	02/2014	Anglesey	*Treponema phagedenis*
13	S3R2	03/2009	Cheshire	*Treponema phagedenis*
14	G2ST24	07/2013	Conwy	*Treponema phagedenis*
15	C2R	06/2009	Gloucestershire	*Treponema phagedenis*
16	Ovine (G179)	2000	Northern Ireland	*Treponema pedis*
17	G3ST1	07/2014	Shrewsbury	*Treponema pedis*
18	G3S45	07/2014	Shrewsbury	*Treponema pedis*
19	G3T1	07/2014	Shrewsbury	*Treponema pedis*
20	G3T7	07/2014	Shrewsbury	*Treponema pedis*

a As determined by 16S rRNA gene phylogenetic analysis.

### *In vitro* antimicrobial susceptibility testing

The MIC/MBC for each antimicrobial was determined using a broth microdilution method as previously described.^6^ One small adjustment was made to the method such that prior to inoculation, bacterial counts were assessed by determining the optical density (OD) of the cultures using spectrometry with wavelength set at 540 nm. The *T. medium/T. vincentii*‐like cultures had an OD of 0.25; the *T. phagedenis*‐like cultures had an OD of 0.43 and the *T. pedis* cultures had an OD of 0.37. This corresponds to 8.75 × 10^7^, 1.14 × 10^8^ and 2.69 × 10^8^ treponemal organisms/mL, respectively.^6^ At this time point, cultures were assessed by phase contrast microscopy to determine that the cultures were alive, of the correct morphology and lacking contaminants. The antimicrobials and their test ranges are listed in Table 2.

**Table 2:** Minimum inhibitory concentrations (MIC) of 10 antimicrobial agents tested against contagious ovine digital dermatitis associated treponemes

Strain no.^a^	Median MIC (mg/L)									
	Penicillin	Amoxicillin	Oxytetracycline	Tilmicosin	Lincomycin	Spectinomycin	Tildipirosin	Tulathromycin	Gamithromycin	Tylosin
1	0.0750	0.5625	3	0.0703	24	12	0.0234	0.2930	0.0469	0.0469
2	0.0375	0.2813	3	0.0234	48	24	0.0234	0.2930	0.0234	0.0234
3	0.0375	0.5625	1.5	0.0117	48	12	0.0469	1.1719	0.0469	0.0234
4	0.0750	0.2813	1.5	0.0234	24	12	0.0469	0.2930	0.0234	0.0469
5	0.0750	0.5625	3	0.0117	24	24	0.0469	1.1719	0.0469	0.0469
6	0.0375	0.1406	0.75	0.0234	24	12	0.0469	0.5859	0.0469	0.0469
7	0.0188	0.1406	0.75	0.0469	12	12	0.0938	0.5859	0.0117	0.0469
8	0.0750	0.2813	0.375	0.0094	12	12	0.0469	0.2930	0.0117	0.0469
9	0.0375	0.1406	0.75	0.1875	24	12	0.0469	0.5859	0.0234	0.1875
10	0.0188	0.1406	0.75	0.0234	6	12	0.0117	0.5859	0.0469	0.0469
11	0.0188	0.2813	0.75	0.0234	3	6	0.0938	0.1465	0.0117	0.0469
12	0.0750	0.1181	0.375	0.0059	6	3	0.0469	0.2930	0.0029	0.0059
13	0.0375	0.1181	0.375	0.375	12	12	0.0234	0.5859	0.0938	0.1875
14	0.0750	0.1181	0.375	0.0938	48	12	0.0234	0.5859	0.0938	0.0234
15	0.0188	0.1406	1.5	0.1875	96	24	0.0469	0.2930	0.0234	0.0938
16	0.0750	0.2813	1.5	0.0234	24	24	0.0234	0.5859	0.0234	0.0938
17	0.0375	0.5625	6	0.0234	48	24	0.0234	0.5859	0.0469	0.0469
18	0.0750	0.5625	3	0.0234	48	12	0.0469	0.5859	0.0234	0.0469
19	0.0750	0.2813	1.5	0.0117	48	24	0.0469	0.5859	0.0234	0.0938
20	0.0750	0.5625	6	0.0117	96	24	0.0234	1.1719	0.0234	0.0938
MIC_90_ b	0.0750	0.5625	3	0.1875	48	24	0.0469	1.1719	0.0469	0.0938

a Isolates 1–5 are *Treponema medium/Treponema vincentii*‐like BDD spirochaetes with antibiotic test ranges (μg/L) of: penicillin G 0.75–0.0059; amoxicillin 2.25–0.0176; oxytetracycline 12–0.0938; tilmicosin 0.375–0.0029; lincomycin 192–1.5; spectinomycin 48–0.375 (Sigma‐Aldrich; Dorset, UK); tildipirosin 0.75–0.0059 (Zuprevo, MSD Animal Health; Milton Keynes, UK); tulathromycin 9.375–0.0732 (Draxxin, Zoetis UK Limited; London, UK); gamithromycin 0.188–0.0015 (Merial LLC; Duluth, Georgia, USA); and tylosin 0.375–0.0029 (Sigma‐Aldrich).

Isolates 6–15 are *Treponema phagedenis*‐like CODD spirochaetes and isolates 16–20 are *Treponema pedis* CODD spirochaetes with test ranges the same as those for *Treponema medium/Treponema vincentii*‐like BDD spirochaetes.

b Cumulative susceptibility results across all treponemes tested are expressed as MIC90, the concentration at which 90% of CODD‐associated treponemes were inhibited.

### Determination of MICs

The MIC for each antimicrobial was taken as the lowest concentration of antimicrobial that prevented growth in the wells observed at the same time points.^6^ Cell growth was determined by comparison of the absorbance measurement immediately after inoculation with the absorbance measured at the late exponential/early stationary phase. All of the absorbance measurements were at 540 nm using a Multiskan microtitre plate reader (Thermo Scientific; Hampshire, UK). The MIC values were taken as the median of three repeat experiments, performed on different days.

### Determination of MBCs

The MBC for each antimicrobial was determined as previously described.^6^

### Determination of MIC_90_ and MBC_90_

The cumulative inhibitory/bactericidal concentration for each antimicrobial tested across all the treponeme isolates was expressed as the concentration at which 90% of CODD‐associated treponemes were inhibited from growing (MIC90) or killed (MBC90).

### Statistical analysis

Differences in MICs between the three different treponeme phylogroups were assessed using the Kruskal–Wallis test. The Kruskal–Wallis test and the nonparametric equality‐of‐medians test were used to compare the MICs for penicillin, oxytetracycline, lincomycin and spectinomycin with previous data,^6^ and for amoxicillin and gamithromycin.^7^ All statistical analyses were conducted using Stata IC 13 (Stata Corp; College Station, TX, USA) and statistical significance was set at *P *<* *0.05.

### Study validation

The MIC microdilution method described in this study was validated by comparing the results produced from four antimicrobials (penicillin, oxytetracycline, lincomycin and erythromycin) incubated with two control microorganisms *T. phagedenis* biotype Reiter and *T. phagedenis*‐like T320A against results previously obtained using a macrodilution method^8^ and also results obtained using a similar microdilution method.^6^, ^7^ The data were also compared statistically using linear regression.

## Results

### Antimicrobial susceptibilities of CODD‐associated treponemes

The individual MIC/MBCs of the antimicrobial agents to each treponeme isolate are summarized in Tables 2 and 3; in this study all isolates showed low MIC/MBCs to all of the antimicrobials tested. Using Table 2, all treponeme groups were most susceptible to gamithromycin and tildipirosin, and least susceptible to lincomycin, spectinomycin and oxytetracycline. The MIC90 for the other five antimicrobials were all relatively low, being <1.0 μg/mL. No bimodal distributions were identified.

**Table 3:** Minimum bactericidal concentrations (MBC) of 10 antimicrobial agents tested against contagious ovine digital dermatitis associated treponemes

Strain no.^a^	Median MBC (mg/L)									
	Penicillin	Amoxicillin	Oxytetracycline	Tilmicosin	Lincomycin	Spectinomycin	Tildipirosin	Tulathromycin	Gamithromycin	Tylosin
1	0.0750	0.5625	6	0.0938	48	12	0.0469	0.5859	0.0469	0.0938
2	0.0750	0.5625	3	0.0469	96	24	0.0469	0.2930	0.0469	0.0938
3	0.0750	1.1250	3	0.0234	48	24	0.0469	1.1719	0.0469	0.0938
4	0.0750	0.5625	3	0.0469	48	24	0.0469	0.5859	0.0234	0.0938
5	0.0750	0.5625	6	0.0234	24	24	0.0469	1.1719	0.0469	0.0938
6	0.0375	0.5625	6	0.0469	48	12	0.0938	0.5859	0.0469	0.3750
7	0.0375	0.5625	3	0.1875	24	24	0.0938	0.5859	0.0234	0.0938
8	0.0750	0.5625	3	0.1875	24	12	0.0469	1.1719	0.0234	0.3750
9	0.0750	0.5625	6	0.1875	96	24	0.0938	0.1172	0.0469	0.1875
10	0.0375	0.2813	6	0.0234	96	12	0.0234	0.5859	0.0469	0.0469
11	0.0189	0.2813	3	0.0234	48	6	0.3750	0.5859	0.0117	0.0469
12	0.0750	0.2813	1.5	0.0117	24	6	0.0469	0.5859	0.0117	0.0117
13	0.0375	0.1181	0.75	0.1875	24	12	0.0234	1.1719	0.0938	0.1875
14	0.0750	0.5625	0.375	0.0938	48	12	0.0234	0.5859	0.0938	0.0938
15	0.0188	0.2813	0.75	0.1875	24	12	0.0469	0.5859	0.0234	0.0938
16	0.0750	0.5625	3	0.0234	48	24	0.0469	0.5859	0.0469	0.0938
17	0.0750	0.5625	6	0.0234	96	24	0.0469	0.5859	0.0469	0.0938
18	0.0750	0.5625	6	0.0469	48	12	0.0469	1.1719	0.0234	0.0938
19	0.0750	0.5625	6	0.0469	48	24	0.0469	1.1719	0.0469	0.0938
20	0.0750	0.5625	6	0.0234	96	24	0.0234	1.1719	0.0469	0.0938
MBC_90_ b	0.0750	0.5625	6	0.1875	96	24	0.0938	1.1719	0.0469	0.1875

a 1–5, *Treponema medium/Treponema vincentii*‐like; 6–15, *Treponema phagedenis*‐like; 16–20, *Treponema pedis*.

b Cumulative susceptibility results across all treponemes tested are expressed as MBC90, the concentration at which 90% of CODD‐associated treponemes were killed.

### Variation in MIC across the different treponeme phylogroups

There was no significant difference in MIC values between the three different phylogroups for five of the seven macrolides (*P* = 0.2), with phylogroup differences for lincomycin and spectinomycin approaching significance (*P* = 0.05). Whilst there was no significant difference between phylogroups in the case of penicillin MIC (*P* = 0.1), in the case of amoxicillin and oxytetracycline, *T. phagedenis*‐like bacteria were more susceptible compared to *T. medium/T. vincentii*‐like and *T. pedis* (*P* = 0.002 and *P* = 0.001, respectively).

### Comparisons with data from previous studies

The MICs for penicillin, oxytetracycline, lincomycin, spectinomycin, amoxicillin and gamithromycin for the 20 isolates investigated here, were not significantly different to those previously reported.^6^, ^7^

### Study validation

The comparison described matched the previous results in all cases except for one antimicrobial (oxytetracycline), which was different by one serial dilution when compared with the macrodilution method.^8^ Linear regression for these comparisons showed strong correlations (*R* = 0.99 *P* < 0.004) indicating the efficacy and reproducibility of this microdilution method.

## Discussion

### Study validation and comparisons with data from previous studies

The methodological validations described reinforce the comparable nature of these current data with previous studies. Comparisons of these current data for penicillin, oxytetracycline, lincomycin and spectinomycin with previous studies do not reveal any statistically significant differences.^6^, ^7^ Therefore, these current data make a valuable contribution to the available data on the *in vitro* antimicrobial susceptibility of these treponemes.

### Antimicrobial use in sheep with CODD

All of the isolates in this study were susceptible (*in vitro*) to all the antimicrobials tested, with gamithromycin, tildipirosin, penicillin, tylosin and tilmicosin demonstrating the lowest MICs and MBCs. This susceptibility, however, may not necessarily be reflected *in vivo*. To date, there have been very few robust *in vivo* studies examining effective treatment. In two clinical studies^4^, ^9^ systemic amoxicillin together with topical chlortetracycline was found to have a clinical cure rate in clinical cases of CODD of approximately 80%. Anecdotally, systemic tilmicosin was also proposed to be an effective treatment for sheep with CODD^10^ and systemic oxytetracycline together with a tylosin footbath were considered to be an effective preventative method.^3^

Currently, no antimicrobial product has a license for CODD in the UK. The antimicrobials studied here were selected to include antimicrobials that already have a license for sheep (penicillin, amoxicillin, oxytetracycline and tilmicosin) together with those that in the authors’ experience are already used (off label) in the sheep industry. Therefore, given this context and these data as a whole, penicillin and tilmicosin would appear to be the most likely candidates for future *in vivo* studies.

This study provides the first detailed examination of the *in vitro* antimicrobial susceptibilities of all three associated phylogroups of treponemes cultured from CODD lesions to antimicrobials. As such, these data provide important *in vitro* information on antimicrobials currently used to treat this disease and should help inform researchers planning further *in vivo* studies when considering which products to include.
